# Chemokine CCL25 Induces Migration and Extracellular Matrix Production of Anulus Fibrosus-Derived Cells

**DOI:** 10.3390/ijms19082207

**Published:** 2018-07-28

**Authors:** Stefan Stich, Anke Möller, Mario Cabraja, Jan Philipp Krüger, Sylvia Hondke, Michaela Endres, Jochen Ringe, Michael Sittinger

**Affiliations:** 1 Tissue Engineering Laboratory, Berlin-Brandenburg Center for Regenerative Therapies, and Department of Rheumatology and Clinical Immunology, Charité-Universitätsmedizin Berlin, corporate member of Freie Universität Berlin, Humboldt-Universitätzu Berlin and Berlin Institute of Health, 10117 Berlin, Germany; anke-moeller-fn@web.de (A.M.); jochen.ringe@charite.de (J.R.); michael.sittinger@charite.de (M.S.); 2 Department of Spinal Surgery, VivantesAuguste-Viktoria-Hospital, 12157 Berlin, Germany; mario.cabraja@vivantes.de; 3 TransTissue Technologies GmbH, 10117 Berlin, Germany; jan-philipp.krueger@transtissue.com (J.P.K.); sylvia.hondke@transtissue.com (S.H.); michaela.endres@transtissue.com (M.E.)

**Keywords:** anulus fibrosus, CCL25, TECK, chemotaxis, differentiation, extracellular matrix, tissue regeneration

## Abstract

Intervertebral disc degeneration is a major source of back pain. For intervertebral disc regeneration after herniation a fast closure of anulus fibrosus (AF) defects is crucial. Here, the use of the C-C motif chemokine ligand 25 (CCL)25 in comparison to differentiation factors such as transforming growth factor (TGF)β3, bone morphogenetic protein (BMP)2, BMP7, BMP12, and BMP14 (all in concentrations of 10, 50 and 100 ng/mL) was tested in an in vitro micro mass pellet model with isolated and cultivated human AF-cells (*n* = 3) to induce and enhance AF-matrix formation. The pellets were differentiated (serum-free) with supplementation of the factors. After 28 days all used factors induced proteoglycan production (safranin O staining) and collagen type I production (immunohistochemical staining) in at least one of the tested concentrations. Histomorphometric scoring revealed that TGFβ3 delivered the strongest induction of proteoglycan production in all three concentrations. Furthermore, it was the only factor able to facilitate collagen type II production, even higher than in native tissue samples. CCL25 was also able to induce proteoglycan and collagen type I production comparable to several BMPs. CCL25 could additionally induce migration of AF-cells in a chemotaxis assay and therefore possibly aid in regeneration processes after disc herniation by recruiting AF-cells.

## 1. Introduction

Back pain is one of the main health problems in industrialized countries [1]. In Germany, 60–70% of the adult population is affected by back pain every year. Thus, back pain is a significant health problem in Germany, which has considerable economic consequences. The costs of treatment for back pain in Germany are 48 billion Euros a year, divided in direct treatment costs (46%) and indirect costs (54%) due to economical production loss [2]. One major source of back pain is intervertebral disc (IVD) degeneration. The presently preferred method of surgical treatment of patients with herniated IVDs remedies the symptoms only temporarily. In 15–25% of operated patients, pain occurs after 1–2 years and 6% of patients have to be re-operated [3]. In herniated discs the leaking nucleus pulposus is removed but ruptures in the anulus fibrosus (AF) remain. That is a major problem that often leads to reherniation. To avoid this, solid barriers are implanted for closure of the remaining gap [4]. The disadvantage is that for fixation these barriers have to be integrated in the vertebral bodies and do not improve regeneration of damaged AF tissue. Also, loosening and dislocation can occur, causing severe pain [5]. Therefore, first experiments in a sheep model towards the closure of an AF defect with natural tissue from the submucosal porcine small intestine were performed and showed improved outcomes compared to untreated defects [6]. Also transplantation of cell-seeded biomaterials for closure of the defect and regeneration of the AF tissue are in focus of research studies [7]. In a two step procedure, autologous cells have to be isolated, expanded, and several weeks later combined with the biomaterial. There are currently no therapies available in clinical routine that support natural regeneration of the AF in a favourable one step procedure.

Another approach to enhance tissue regeneration and subsequent AF closure is the application of growth factors. Already in 1991, Thompson et al. reported effects of various growth factors on cell proliferation and proteoglycan (PG) synthesis in organ cultures of canine IVD cells. Extracellular matrix (ECM) PGs play an important functional role due to their high water-binding capacity in the disc [8]. Factors such as BMP2 [9], BMP7 [10], and BMP14 [11] led to increased PG synthesis in various animal cell cultures in vitro or to an increase in AF tissue thickness in animal models. In a comparative in vitro study in which various BMPs were adenovirally transduced into bovine AF cells, almost all BMPs were found to be effective in increasing PG content and collagen type II formation. BMP13 was found to be the most effective BMP [12]. In human cells, an increase in PG content could also be determined in vitro following addition of BMP7 to AF cells [13].

Another group of interesting factors are chemokines. However, so far, they were not discussed in context of AF cells or AF closure. As chemotactic cytokines they are able to induce cell migration and could possibly initiate AF cell recruitment from intact surrounding AF tissue into the defect area of the herniated discs and lead to an induction of tissue regeneration. Among this group of factors, C-C motif chemokine ligand (CCL)25 also known as thymus—expressed chemokine (TECK), has shown a high recruitment potential on mesenchymal stromal cells [14,15], which are often discussed in context of IVD regeneration [16].

Platelet lysate from platelet-rich plasma (PRP) contains a concentrate of various numbers of growth factors and cytokines. Among them are different members of the TGFβ superfamily, such as BMP2, BMP4, BMP7, BMP12, TGFβ2 and TGFβ3, known to have an impact on synthesis of IVD and cartilage [17] ECM synthesis. Platelet lysate from PRP is also known to induce glycosaminoglycan production in AF-derived cells [18].

Focussing on our overall aim to establish a regenerative approach for AF closure, based on implantation of biomaterials supplemented with growth factors and/or cell recruiting factors like chemokines, the aim of our current study was to compare the effect of BMP2, -7, -12, -14, TGFβ3, which were found as a component in PRP [17], CCL25 and PRP-derived platelet lysate on the formation of extracellular AF matrix, in a 3D high-density cell culture assay. Formation of collagens and PGs by human AF cells will be analyzed by histology and immunohistochemistry. Moreover, for the first time, using a Boyden-chamber chemotaxis assay, we want to analyse migratory effects of CCL25 on human AF cells. Besides the migratory effect, the main question of this study is if CCL25 is able to support cell matrix production of AF in vitro comparable to known factors of the TGFβ-superfamily.

## 2. Results

### 2.1. Chemotaxis Assay

**Figure 1:**
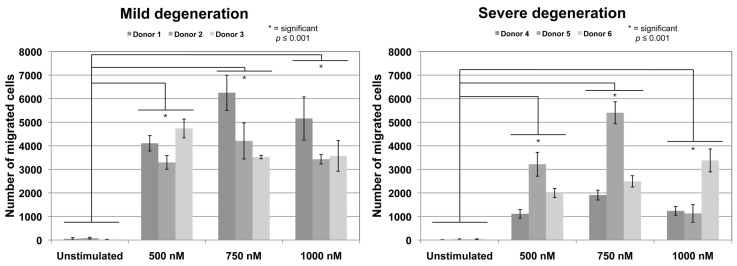
Chemotaxis assay. Migration of lumbar AF cells derived from donors with mild disc degeneration (donors 1–3) and severe disc degeneration (donors 4–6) (all measured in triplicates; error bars: standard deviation). Significant increased migration (* *p* ≤ 0.001) was found in all CCL25 concentrations (500, 750, and 1000 nM) compared to unstimulated controls. There were no significant differences between concentrations. Also, no significant differences between cells form mild and severe degenerated AF for same CCL25 concentrations were detected.

To evaluate the potential of CCL25 to induce cell migration of AF-derived cells, concentrations of 500, 750 and 1000 nM CCL25 were applied in the Boyden-chamber chemotaxis assay. Cells derived from mild (Pfirmann-score II–III) and severe (Pfirrmann-score IV–V) degenerated human AF tissues of 3 independent donors each were tested in triplicates. All concentrations induced a significant (*p* ≤ 0.001) increase of migrated cells compared to cells in unstimulated control groups without CCL25 (Figure 1). In general, absolute cell numbers of migrated cells were slightly higher for cells from donors with mild degenerated AF. For cells derived from tissues of donors 1 and 2, 750 nM showed the highest dose-dependent migratory effect. For cells derived from donor 3, 500 nM revealed the highest effect. Cultures derived from severe degenerated AF tissue of donors 4 and 5 demonstrated the highest number of migrated cells at 750 nM while cells from donor 6 showed the highest migration at 1000 nM CCL25.

### 2.2. Scratch-Wound Assay

**Figure 2:**
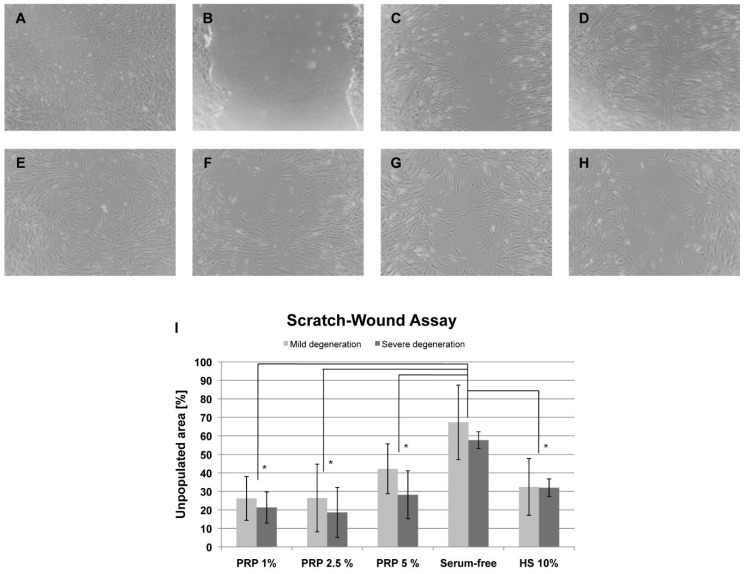
Scratch-wound assay. Exemplarily shown cell layer before the scratch (**A**), directly after the scratch (**B**), and the closing scratch-wound after 24 h (**C**) and 48 h (**D**) using medium containing 2.5% PRP reveal the closure of the gap. For comparison, exemplary images of the closing gap after 48 h were given for 1% PRP (**E**), for 5% PRP (**F**), 10% human serum supplemented medium (**G**), and for serum-free medium (**H**) (magnification of all images 40×). The unpopulated area at 48 h as a percentage of the area directly after the scratch (**I**) reveals the lowest open area with a concentration of 2.5% PRP for AF cells derived from mildly and severely degenerated AF tissue (* *p* < 0.05). Values of the unpopulated area were given as mean values of 3 donors with mild and severe disc degeneration each. Cells of each donor were measured in triplicates (error bars: standard deviation).

To determine a migratory effect of PRP-derived platelet lysate on AF cells a scratch-wound assay was performed with PRP concentrations of 1%, 2.5%, and 5% in standard cultivation medium as serum replacement. The assay was performed with the cells from the same donors as the chemotaxis assay (mild degenerated AF tissues: donors 1–3; severe degenerated AF tissues: donors 4–6) and each measured in triplicates. The scratch was applied in a confluent layer of AF cells (Figure 2A,B). Closure of the gap by cell growth was documented after 24 h (Figure 2C) and 48 h (Figure 2D). Results are exemplarily shown for 2.5% PRP (Figure 2A–D). For comparison, exemplary images of the closing gap after 48 h were given for 1% PRP (Figure 2E), for 5% PRP (Figure 2F), 10% human serum supplemented medium (Figure 2G), and for serum-free medium (Figure 2H). Mean results from TScratch software to determine the percentage of the open area after 48 h compared to the scratch at 0 h revealed that 1% (mean of 23.8% open space) and 2.5% of PRP (mean of 22.5% open space) showed the most efficient closure of the gap. Medium with 5% PRP (mean of 35.2% open space) was slightly less effective than standard medium with 10% human serum (mean of 32.2% open space). Serum-free medium left an open space of 62.5% in mean. All PRP concentrations and the 10% human serum group were significantly lower than the serum-free group for cells derived from mild and severe degenerated tissues (* *p* < 0.05). There were no significant differences between mild and severe groups for the same medium (Figure 2I).

### 2.3. Factor Screening Assay

Since the chemotaxis assay and the scratch-wound assay revealed no significant differences in cell migration potential between mild and severe degenerated cells, the factor screening in 3D high-density AF cell cultures (pellets) was performed with cells of three donors with different disc degeneration grades according to Pfirrmann Score (donor 7—mild degeneration (grade II); donor 8—mild-severe degeneration (grade III); donor 9—severe degeneration (grade IV)). Pellets were induced with medium supplemented with BMP2, BMP7, BMP12, BMP14, TGFβ3 or CCL25 in concentrations of 10, 50 or 100 ng/mL.

**Figure 3:**
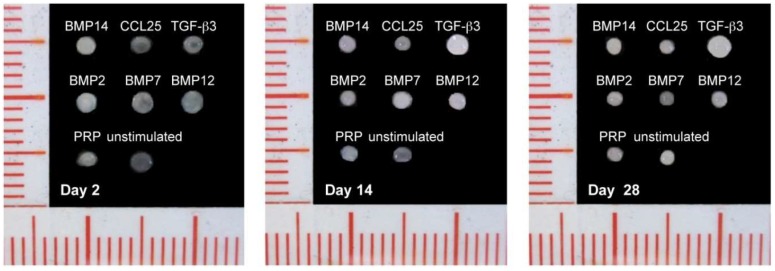
Pellet formation of AF cells (exemplarily donor 7, 100 ng/mL supplemented factor or 2.5% PRP or unstimulated) showed an increase of stiffness and a decrease in size, except of TGFβ3 stimulated pellets. There, also an increase of size was visible (scale: scale line distance = 1 mm; main scale line distance = 5 mm).

Over the 28 days of differentiation in the pellet differentiation assay the size of the pellets was shrinking and the solidity was changing (Figure 3). At day 2 after stimulation, the pellets were very soft with a diameter of 1.8–2.0 mm. Haptic impressions indicated an increase in solidity/density of the pellets over the time of cultivation. All pellets were shrinking over the 28 days (1.5–1.7 mm at day 14; 1.3–1.5 mm at day 28. The exception was TGFβ3, where size (1.9 mm at day 2; 2.0 mm at day 14; 2.3 mm at day 28) and solidity were both increasing.

**Figure 4:**
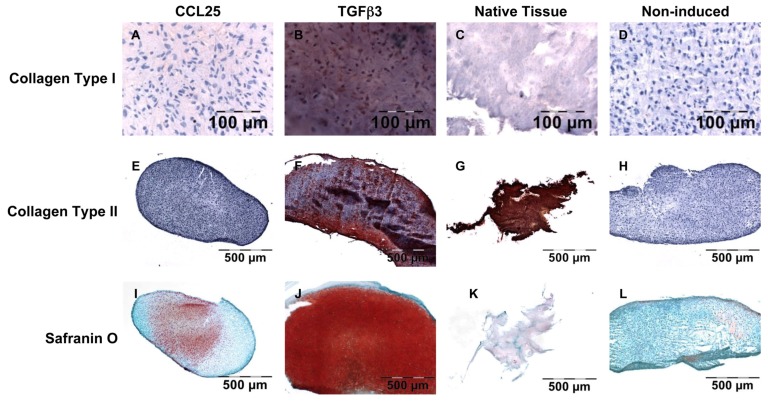
Histological and immunohistochemical staining. Exemplary visualization of collagen type I immunohistochemical staining of CCL25 induced (**A**), TGFβ3 induced (**B**) AF cells in pellet cultures; native AF tissue (**C**), and non-induced (**D**) AF cells in pellet cultures; collagen type II immunohistochemical staining of CCL25 induced (**E**), TGFβ3 induced (**F**) AF cells in pellet cultures; native AF tissue (**G**), and non-induced (**H**) AF cells in pellet cultures; safranin O staining of CCL25 induced (**I**), TGFβ3 induced (**J**) AF cells in pellet cultures; native AF tissue (**K**), and non-induced (**L**) AF cells in pellet cultures.

**Figure 5:**
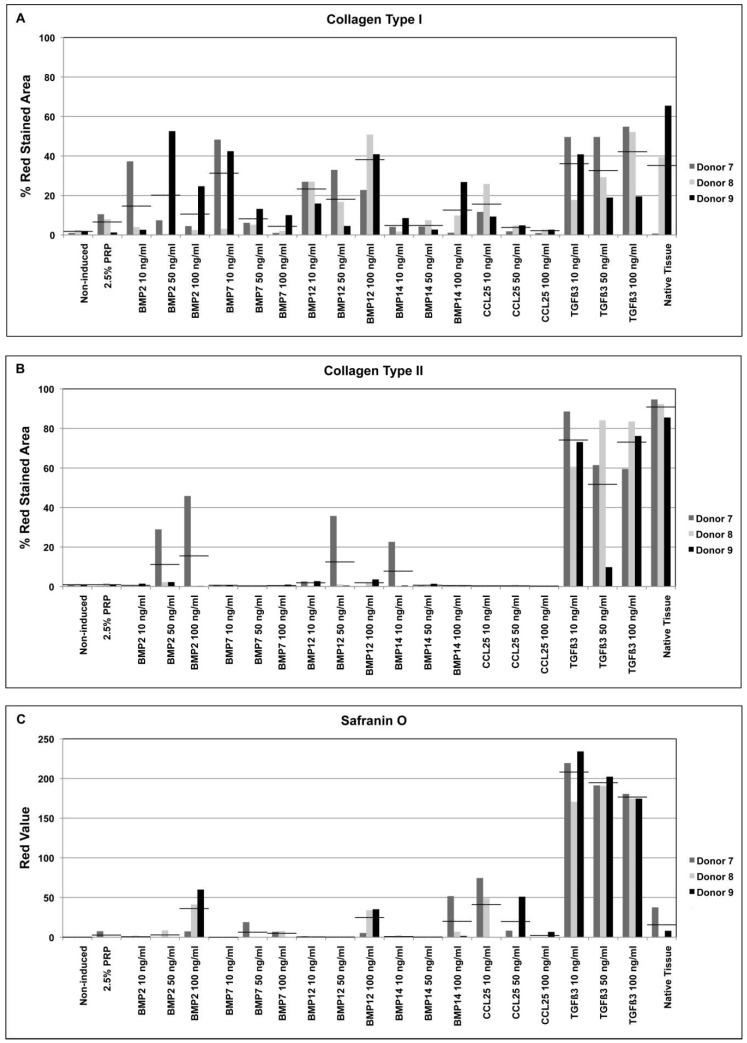
Histomorphometric analysis of collagen and proteoglycan formation. Collagen type I (**A**) and type II (**B**) stainings were analyzed histomorphometrically and the red stained areas calculated and given as a percentage of the sample. Histomorphometric analysis of safranin O staining determined the red value of the samples (**C**). Average values of the 3 donors were given as horizontal bars (___).

As shown by immunohistochemical staining, 28 days of induction with CCL25 led to a weak formation of collagen type I (Figure 4A), while TGFβ3 induced samples revealed an intense staining (Figure 4B) both compared to native tissue (Figure 4C) and non-induced controls (Figure 4D). Histomorphometric analysis of the immunohistochemical staining revealed that collagen type I was formed in nearly all TGFβ3 induced pellet cultures, reaching levels similar to native tissue samples in all supplemented concentrations (Figure 5). Also, the cultures induced with 10 ng/mL BMP7 and 100 ng/mL BMP12 demonstrated a similar result. CCL25 (10 ng/mL), BMP14 (100 ng/mL) and BMP2 treatment resulted in slightly lower histomorphometric values, while the induction with platelet lysate from PRP was even lower but still above non-induced samples. For PRP supplementation of the medium, 2.5% were used. This value showed the best results in the scratch-wound assays indicating an appropriate concentration for future cell recruitment-based approaches.

Formation of collagen type II rich matrix was not seen in CCL25 induced samples (Figure 4E) and only measurable amounts were observed in TGFβ3 induced pellets in all concentrations (Figure 4F) in a comparable level to native tissue (Figure 4G). Non-induced controls showed no collagen type II production (Figure 4H). Histomorphometric analysis confirmed this data. A further induction was only seen in 50 and 100 ng/mL BMP2, 50 ng/mL BMP12, and 10 ng/mL BMP14 induced pellets of cells of donor 7 (Figure 5).

Safranin O staining demonstrated an enhanced PG production in CCL25 induced samples comparable to native tissue (Figure 4I,K). An even stronger PG production was found in TGFβ3 induced pellets (Figure 4J). The massive production of safranin O by TGFβ3 was confirmed in all 3 concentrations. Non-induced pellets revealed no PG formation by safranin O staining (Figure 4L). Furthermore, BMP2, BMP12, and BMP14 induced samples showed an increased safranin O staining of PGs in 100 ng/mL induced samples (Figure 5). Taken together PRP and BMP7, could only induce collagen type I production, whereas BMP2, BMP12, BMP14, and CCL25 were able to induce collagen type I and PG synthesis. Only TGFβ3 induced all 3 tested ECM components. However, PG production was up to 7 times higher compared to native AF tissue.

## 3. Discussion

In our study, we could demonstrate for the first time, that the chemokine CCL25 is able to induce migration of human AF-derived cells. Furthermore, CCL25 could stimulate PG and collagen type I production in our in vitro high-density 3D micromass pellet culture system. Our targets to demonstrate ECM production in our pellet system were collagen type I and type II since they are the most prominent in the AF ECM. These two types of collagen fibrils are making up approximately 70% of the dry weight of the AF with collagen type I more prominent in the outer part of the AF and collagen type II in the inner part [19,20]. Furthermore, safranin O staining was used to demonstrate PGs, making about 20% of the wet weight of the AF [20]. In the cell migration assay and the scratch assay AF cells from mild and severe degenerated tissues were used with only marginal differences in the results. This is in accordance with other studies where proliferation, migration, and ECM formation of AF cells derived from mild and severe degenerated AF tissues were analyzed [21]. A different study analyzed the PG/collagen ratio in AF tissues from different stages of IVD degeneration and donor ages [22]. Here, was also no correlation of the measured results to degeneration grade of the IVD and donor age.

Considering the PG production, BMP2, BMP12, and BMP14 showed similar results compared to CCL25 and native AF tissue. Only TGFβ3 enabled up to 7 fold higher PG content in the extracellular matrix than in native AF tissue. Since in the AF the PG content is only 20% of the wet weight [20], the 7 fold higher induction seems to outbalance the concentration of collagens and PG in TGFβ3 induced samples. BMP7 and platelet lysate derived from PRP were only able to induce a small amount of PG production.

In this study, cells were cultivated with 2.5% platelet lysate of PRP. This led to an increased proliferation and migration of AF cells in the scratch-wound assay, but only to a minimal induction of PGs in the pellet assay. Other studies with bovine cells used higher amounts of 25–50% platelet lysate to demonstrate PG induction in AF cell cultures [18]. In alginate 3D cultures with porcine cells, 10% PRP was used to effectively stimulate AF cells for matrix and collagen production [23]. The use of 2.5% platelet lysate might have been optimal for AF cell proliferation and migration but not for PG production. Similar results were achieved in a study with 5% PRP in a scaffold-based 3D AF model. An enhanced proliferation of the cells was induced but no production of PGs and collagens were detected [21]. For PRP in our study, only a minor collagen type I induction compared to the other tested factors was detectable in our 3D pellet system, whereas collagen type II was not visible similar to non-induced cells. The absence of collagen type II secretion was also observed in BMP7 and CCL25 treated pellets. BMP2 stimulation resulted in an increased collagen type II staining for cells of one donor treated with 50 and 100 ng/mL. The same cells also responded to 50 ng/mL BMP12 and 10 ng/mL BMP14 inductions. Only TGFβ3 stimulation revealed a similar stained area compared to native tissues indicating collagen type II production.

In contrast to the weak collagen type II staining, collagen type I staining demonstrated larger stained areas. Most effective collagen type I production of BMP2 treated pellets was found in concentrations of 10 and 50 ng/mL dependent on the donor. BMP7 showed the highest collagen type I induction using 10 ng/mL. BMP12 and BMP14 presented their largest stained areas of collagen type I using 100 ng/mL induction, with BMP12 achieving a similar level compared to native tissue samples. All three concentrations of TGFβ3 resulted in similar efficient collagen type I induction. Besides the factors that are known for collagen induction [12], also the chemokine CCL25 was able to do so in a ratio similar to BMP14 when supplemented in a concentration of 10ng/mL.

Since CCL25 was able to induce collagen type I and PG production in AF cells it might be an interesting candidate for our overall aim to establish a regenerative approach for AF closure, based on implantation of biomaterials supplemented with growth/differentiation factors and/or cell recruiting factors. With the capability to recruit AF cells and then after recruitment to induce ECM synthesis in these cells an additional growth/differentiation factor might not be needed. A corresponding receptor of CCL25 is CCR9. It was already demonstrated that CCR9 is expressed in AF cells on gene expression level [24], but CCL25 could not induce a significant migration in that study. In our study, the CCL25 induced migration was detectable in cells derived from severe and mild degenerated AF tissue in a range of 500–1000 nM. The use of CCL25 to induce migration in other mesenchymal cells (bone marrow derived mesenchymal stem/stroma cells [15], periosteum derived mesenchymal progenitor cells [14] or mesenchymal progenitors derived from subchondral cortico-spongious bone [25]) was already tested in concentrations of 1,10, 50,100, 250, 500, 750, and 1000 nM. There, results showed that an optimal dose-dependent migration with CCL25 was always induced in concentration between 500–1000 nM (mesenchymal stem/stromal cells: 1000 nM, periosteum derived progenitor cells: 500 nM, progenitors from cortico-spongious bone: 1000nM). Lower concentrations led to migration but were barely statistical significant compared to unstimulated cells. Therefore, we focused our experiments on the target concentrations 500, 750, and 1000 nM. For the other factors used in our pellet differentiation assay an inducing migratory effect on AF-derived cells is not described in the literature. A recent test demonstrated that TGFβ3 was not able to induce the migration of these cells in vitro [21]. The chemotactic effects of different BMPs like the used BMP2, BMP7, and also TGFβ on articular chondrocytes were already evaluated. No significant increase of migration was found using the three mentioned growth factors [26].

Nevertheless, besides the impact on PG and collagen production members of the TGFβ-superfamily are in discussion. Besides their ability to induce collagen and PG production in AF cells [9,10,11,12,13] they may also have an impact on AF ossification. It was reported that in concentrations of 1 µg/mL BMP2 and TGFβ3 induce ossification in the AF in an intervertebral disc rabbit explant model. Therefore, these factors were also suggested to be used in similar concentrations to induce osseous fusion of intervertebral discs [27]. When transferring the results of our study for AF regeneration, a supplementation of these two factors, especially as seen for TGFβ3 may lead to an ossification of newly formed tissue or the surrounding AF. In human clinical applications, BMP2 is used for spinal fusion [28]. Nevertheless, the use of BMP2 for this application is also discussed due to occurring side effects. Depending on the spinal area, the use of BMP2 may lead to ectopic bone formation or inflammation causing severe dysphagia and airway compromise [29]. BMP7 combined with an autograft was tested in clinical trial studies but could not demonstrate a significant increase of spinal fusion compared to an autograft alone without BMP7 [30,31]. Also, BMP14 is considered for spinal fusion. In a rabbit model BMP14 in combination with a Healos carrier (cross-linked type I collagen with hydroxyapatite coating) showed a significantly higher spinal fusion rate than only an autograft control group [32]. Two clinical trials considering the safety, tolerability and the preliminary effectiveness of rhBMP14 are ongoing [33]. Therefore for an application in a regenerative approach for the closure of AF defects these mentioned factors might not be the best choice. The impact of CCL25 on AF ossification is unknown so far. Also the application of PRP might be of interest. Despite showing only a weak induction of collagen type I PRP can be prepared from the patient’s own blood enabling an autologous application.

Taken together, here we demonstrate for the first time that the chemokine CCL25 is able to stimulate the migration of AF cells and, surprisingly, to induce PG and collagen type I production in a 3D micromass pellet culture system of human AF derived cells on a similar level compared to several BMPs. Considering the overall aim of regenerating AF defects based on implantation of biomaterials supplemented with growth/differentiation factors and/or cell recruiting factors, CCL25 provides in vitro all recommended abilities. With the capability to recruit AF cells (migration assay) and after recruitment to induce ECM synthesis (3D pellet model) in these cells an additional supplementation or a combination of growth/differentiation factors might not be needed.

Therefore, CCL25 represents an interesting candidate for factor induced AF regeneration and closure of AF defects in vivo. Further studies for CCL25 in in vivo approaches should be performed to prove the capability of CCL25 to recruit cells from healthy AF tissue to damaged tissue areas and further to induce these cells to matrix formation after recruitment. In that case, a factor release system might be of interest to provide a proper CCL25 distribution with a high released concentration in the cell recruitment phase and a constant lower release during the collagen and PG synthesis phase.

## 4. Materials and Methods

### 4.1. Preparation of Human Serum and PRP

Human serum was prepared from full coagulated blood bags of healthy donors (German Red Cross, Berlin, Germany). Serum was centrifuged at 3500× *g* for 10 min without a brake. Supernatant was inactivated for 30 min at 56 °C. The serum was stored at −20 °C until used.

PRP (German Red Cross) (*n* = 5) of normal, healthy donors was pooled and frozen at −20 °C. For preparing the platelet lysate an aliquot of pooled PRP was thawed at 4 °C and centrifuged at 1600× *g* at 4 °C for 10 min. Supernatant was frozen again at −20 °C. After an additional thawing and centrifugation, the freezing of the supernatant and the thawing procedure was repeated for a third time. The final supernatant containing the platelet lysate was used in experiments.

### 4.2. Isolation and Cultivation of Anulus Fibrosus Cells

Cells were isolated from native human lumbar AF tissue samples of different degeneration grades provided by volunteering donors undergoing spinal surgery. All donors gave their informed consent for inclusion before they participated in the study. The study was conducted in accordance with the Declaration of Helsinki. The study was approved by the ethics committee of the Charité-Universitätsmedizin Berlin (project identification code EA2/102/15 approved 06/08/2015). Scoring of degeneration grades was performed according to MRI images using Pfirrmann score [34]. Tissue samples were rinsed and a small fragment of 0.3 cm × 0.3 cm was separated, embedded in a cryomold containing TissueTek (Sakura Finetek, Staufen, Germany), and frozen in liquid nitrogen. The frozen tissue was stored at −80 °C for histological and immunohistochemicalstainings. The remaining tissue was minced in 1 mm^2^ sized fragments and placed in a spinner flask containing 30 mL isolation medium (Dulbeccos modified Eagle Medium (1 g/L glucose) (Biochrom, Berlin, Germany), 10% human allogenic serum, 1% penicillin/streptomycin (Biochrom), 10,000 U collagenase CLS II (Biochrom), 30 U collagenase P (Roche, Mannheim, Germany), and 1000 U hyaluronidase (Sigma-Aldrich, Munich, Germany)). After digestion over night cells were rinsed and the remaining fragments were removed. Cells were seeded in cell culture flasks with a density of 10,000 cells/cm^2^ and cultivated with growth medium (Dulbeccos modified Eagle Medium (1 g/L glucose), 10% human allogenic serum, 1% penicillin/streptomycin, 2 ng/mL human fibroblast growth factor 2 (Peprotech, Hamburg, Germany)). At 80–90% of confluence cells were harvested with trypsin/EDTA and seeded with a density of 5000 cells/cm^2^. Cultivation was performed up to passage 4.

### 4.3. Chemotaxis Assay

Chemotaxis assays to analyse the recruiting potential of the chemokine CCL25 (Peprotech) on AF cells were performed using 96-multiwell ChemoTx plates with 8 µm pore size (Neuroprobe, Gaithersburg, MD, USA). Cells of donors with mild (Pfirmann-score II–III) and severe (Pfirmann-score IV–V) disc degeneration grades (*n* = 3 each) were used for chemotaxis analysis. The chemokine CCL25 (Peprotech) was diluted in serum-free medium (Dulbeccos modified Eagle Medium (1 g/L glucose) (Biochrom), 1% penicillin/streptomycin (Biochrom), 0.5% bovine serum albumin (Sigma-Aldrich)) in concentrations of 500, 750, and 1000 nM and 37.5 µL were filled in triplicates in the lower chamber. The membrane was placed on top and 30,000 cells in a volume of 40 µL serum-free medium were placed on the upper side of the membrane. After 20 h of incubation non-migrated cells on the upper side of the membrane were removed and migrated cells on the lower side were stained using the Hemacolorstaining Kit (Merck, Darmstadt, Germany). Stained cells were counted in two microscopic images (40× magnification) and extrapolated to the whole well. Statistical significances of migration for stimulated cells with 500, 750, and 100 nM compared to unstimulated cells, between concentration and between cells from mild and severe degenerated tissue were calculated. Normal distribution was determined using the D’Agostino and Pearson omnibus normality test. Statistical significance was analyzed using *t*-test of GraphPad Prism v5 software (GraphPad Software, La Jolla, CA, USA). Differences were considered significant at *p* ≤ 0.001.

### 4.4. Scratch-Wound Assay

The scratch-wound assay was used to evaluate an appropriate PRP concentration for cell migration. AF cells of donors with mild and severe disc degeneration grades (*n*=3 each) were used. Cells at passage 4were seeded in triplicates in 24-well plates with a density of 15,000 cells/well in a volume of 500 µL and cultivated until confluence. With the tip of a pipette a scratch was made in the confluent layer cutting cell layers in halves. Growth medium containing 10% human serum, serum-free medium with ITS^+^ supplement (Sigma-Aldrich) and medium with either 1%, 2.5% or 5% of activated PRP were used to replace the standard growth medium. Every medium was also supplemented with the anti-coagulant acid citrate dextrose (ACD) (Sigma-Aldrich) in a ratio of 1:1 to avoid coagulation of PRP. Cells were allowed to grow to replace the empty space left by the scratch for 48 h. Photographs were taken directly after applying the scratch, 24 and 48 h later. The ratio between the residual open area after 48 h and the initial prepared scratch area was calculated and given as percentage of the open area after the initial scratch [35]. Normal distribution was determined using the D’Agostino and Pearson omnibus normality test. Statistical significances of migrated cells compared to serum-free controls and between cells from mild and severe degenerated tissue were calculated using *t*-test of GraphPad Prism v5 software. Differences were considered significant at *p* ≤ 0.001.

### 4.5. Factor Screening Assay

A micromass pellet system was used to determine a factor enhancing AF matrix formation. After the third passage, AF cells of three individual donors were washed twice with serum-free differentiation medium (Dulbeccos modified Eagle Medium (4.5 g/L glucose) (Biochrom), 2% HEPES (Biochrom), 1% penicillin/streptomycin (Biochrom), 1% ITS^+^ supplement (Sigma-Aldrich), 0.1 µM dexamethasone (Sigma-Aldrich), 1 mM sodium pyruvate (Sigma-Aldrich), 0.17 mM ascorbic acid (Sigma-Aldrich), 0.35 mM proline (Sigma-Aldrich)) and 250,000 cells were transferred to 15 mL tubes. To the serum-free medium BMP2, BMP7, BMP12 (growth differentiation factor 7 (GDF7)), BMP14 (GDF5), TGFβ3 or CCL25 (all Peprotech) were added in concentrations of 10, 50 or 100 ng/mL. Additionally, samples with 2.5% PRP and non-stimulated controls without any other factor were prepared. Six samples of each factor and each concentration were prepared. Over all, 120 pellet cultures in 20 different media were prepared for cells of each donor. The pellets were cultivated for 28 days with a medium change three times a week. Samples were taken at day 2, day 14, and day 28. Each pellet was placed in a cryomold and embedded in TissueTek (Sakura Finetek). After freezing in liquid nitrogen the pellets were stored at −80 °C until used for histological and immunohistochemical analysis.

### 4.6. Histological and Immunohistochemical Analysis of Anulus Fibrosus Matrix Production

To demonstrate collagen production immunohistochemical staining were carried out. To show extracellular matrix production safranin O staining was performed to determine PG formation. In brief, frozen samples in TissueTek were sectioned with a thickness of 6 µm and transferred to a glass slide. For the verification of collagen type I and type II production, immunohistochemical detection was performed using primary monoclonal mouse anti-human antibodies against collagen type I (BioRad, Puchheim, Germany) and collagen type II (Merck). For visualisation the DAKO EnVision Kit (DAKO, Hamburg, Germany) was used according to the manufacturer’s protocol. In brief, the cryosections were incubated with primary antibodies for 40 min. The secondary horseradish peroxidase labeled goat anti-mouse antibody solution was also applied for 40 min. Last, the samples were incubated with the substrate AEC for 10 min and counterstained for 10 min with hematoxylin (DAKO). For safranin O staining of sulphated PGs the samples on the glass slides were stained with 0.7% safranin O (Sigma-Aldrich) in 66% ethanol for 30 min. For counterstaining, cell nuclei were stained with 0.2% Fast Green FCF (Sigma-Aldrich) solved in 75% acetic acid for 1 min. Photographs of the staining were taken and analyzed histomorphometrically. For the determination of red values for safranin O staining and the red stained areas of immunohistochemical collagen types I and II staining a histomorphometric analysis was performed as described before [36].

## Abbreviations

ACD	Acid citrate dextrose
AF	Anulus fibrosus
BMP	Bone morphogenetic protein
CCL	C-C motif chemokine ligand
ECM	Extracellular matrix
GDF	Growth differentiation factor
IVD	Intervertebral disc
PG	Proteoglycan
PRP	Platelet-rich plasma
TECK	Thymus—expressed chemokine
TGF	Transforming growth factor
